# Conservative treatment and follow-up of vaginal Gartner’s duct cysts: a case series

**DOI:** 10.1186/s13256-016-0936-1

**Published:** 2016-06-02

**Authors:** Salete S. Rios, Lara Cristina R. Pereira, Carla B. Santos, Ana Carolina R. Chen, Juliana R. Chen, Maria de Fátima B. Vogt

**Affiliations:** Obstetrics and Gynecology Department, School of Medicine, University of Brasilia DF, Campus Universitário Darcy Ribeiro, Brasília, DF 70910-900 Brazil

**Keywords:** Vaginal Gartner’s cyst, Conservative treatment

## Abstract

**Background:**

In women, during embryologic development, the paired Müllerian (paramesonephric) ducts fuse distally and develop into the uterus, cervix, and upper vagina. If the Wolffian ducts persist in vestigial form, they can lead to Gartner’s cysts, mainly located in the right wall of the vagina. This is one of the few studies of Gartner’s cysts with a series of consecutive cases over a long period of time who were exclusively subject to clinical observation. Although Gartner’s cysts are found in approximately 0.1 to 0.2 % of women, controversy exists regarding the course of action to be taken.

**Case presentation:**

We describe the cases of four women who were 38-years old, 53-years old, 37-years old, and 49-years old at their first appointment and who were of mixed ethnicity, mixed ethnicity, black, and mixed ethnicity respectively. The follow-up of these patients ranged from 2 to 17 years. In these four cases the location of the cysts was the right wall of the vagina. Transvaginal ultrasound was the test of choice for diagnostic confirmation. In the cases presented in this study, the women were asymptomatic and chose to be observed clinically.

**Conclusions:**

This is the first study reporting long-term clinical observation of these lesions. This study shows that conservative treatment can be a safe option for asymptomatic patients with vaginal Gartner’s duct cysts.

## Background

The internal urogenital tract is derived from two sets of ducts: the Wolffian ducts (mesonephric) and the Müllerian ducts (paramesonephric). These ducts are present in both sexes. In women, during the eighth week of embryologic development, the paired Müllerian (paramesonephric) ducts fuse distally and develop into the uterus, cervix, and upper vagina. In addition, the Wolffian ducts regress. If the ducts persist in a vestigial form, they can form Gartner’s cysts. These cysts are mainly located in the right anterolateral wall of the vagina and less commonly in the lateral walls of the uterus [1, 2]. The imperfect development of Wolffian ducts can also result in urogenital abnormalities, such as changes in the metanephric urinary system [1, 3]. Cases of ectopic ureter, unilateral renal dysgenesis, and renal hypoplasia have also been reported in association with Gartner’s cysts [1, 4]. Most of these lesions are asymptomatic, but they may be accompanied by infections, bladder dysfunction, abdominal pain, vaginal discharge, and urinary incontinence due to extrinsic compression of the bladder neck [3]. True Gartner’s duct cysts are typically located along the anterolateral wall of the proximal third of the vagina. In contrast, Bartholin’s cysts are typically located in the posterolateral wall of the lower third of the vagina and are associated with the labia majora [1, 5]. The differential diagnosis might include Bartholin’s gland cyst, uterine prolapse, cystocele, rectocele, enterocele, urethral diverticulum, endometriosis, and malignant growth, among others [6]. Although vaginal cysts are found in approximately 1 to 2 % of women and Gartner’s duct cysts comprise approximately 10 % of vaginal benign cysts [1, 7], there is still some controversy regarding which course of action should be taken. This is the first study reporting long-term clinical observation of these lesions.

## Case presentation

### Case 1 – follow-up for 17 years

**Fig. 1:**
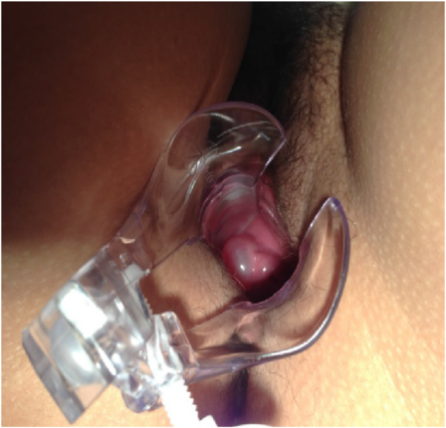
Paracervical cystic lesion with translucent content on the right wall of the vagina, measuring approximately 2 × 2 cm

A 38-year-old woman of mixed ethnicity was initially referred to our service at 21 years of age for evaluation before starting contraceptive use. She reported menarche at 13, first sexual intercourse at 21 and regular cycles. No abnormalities were observed during a breast examination. A speculum examination revealed an epithelialized cervix and the presence of a paracervical cystic lesion on the right wall of her vagina, painless to manipulation, measuring approximately 2 × 2 cm, with translucent content and soft consistency (Fig. 1). An ultrasound revealed a cystic lesion. She continued regular annual monitoring. She became pregnant at age 32 and delivered by cesarean section. The cyst remained approximately 1.5 to 2.0 cm in size and did not change during pregnancy. She has received follow-ups since that time, and the Gartner’s cyst has exhibited no change in its characteristics.

### Case 2 – follow-up for 13 years

**Fig. 2:**
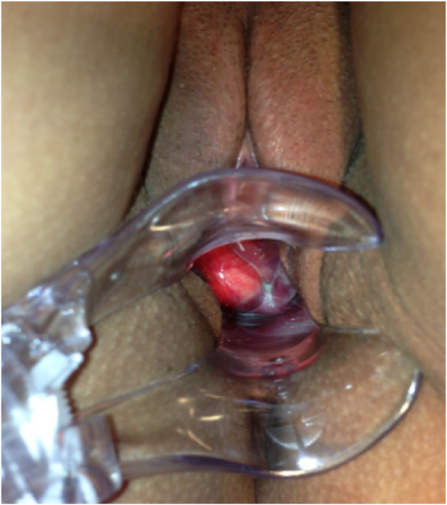
Cystic lesion in right wall of the vagina with translucent content, but with exuberant vascularization

A 53-year-old G3P3 woman of mixed ethnicity sought our service at 40 years of age for a routine visit. She had no significant history of medical illness. Upon speculum examination, a cystic lesion in the right wall of her vagina with translucent content and extensive vascularization was found (Fig. 2). A transvaginal ultrasound revealed a hypoechoic nodule in the vaginal fornix measuring 4.2 × 3.8 × 2.1 cm, with thick content, and was consistent with a cyst. Removal was advised due to the thick content and exuberant vascularization, but she declined. She has been followed to date with no alterations.

### Case 3 – follow-up for 5 years

**Fig. 3:**
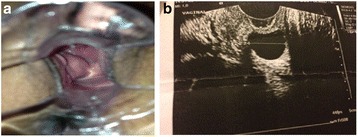
**a** Cystic lesion in the right wall of the vagina close to the fornix. **b** Ultrasound showed a thin-walled cyst with anechoic content on the right posterolateral wall of the vagina

A 37-year-old black woman presented to our service at age 32 for a Pap test. Her obstetric/gynecological history was significant for menarche at age 13, first sexual intercourse at 15, and regular menstrual cycles. Her pregnancies included one natural delivery, one tubal and one anembryonic pregnancy. She had a left salpingectomy. She also had a history of urethral stricture that led to recurrent urinary tract infections (UTIs). Her family history notes an aunt with unspecified urethral alterations. Her speculum examination revealed a healthy cervix and the presence of a cyst measuring approximately 2.5 × 2 cm in the proximal third of the right wall of her vagina (Fig. 3a). Touch revealed a lesion with cystic consistency. She underwent an ultrasound that showed a thin-walled cyst with anechoic content on the right posterolateral wall of her vagina (Fig. 3b). The findings, along with measurements of 2.2 × 1.8 × 1.9 cm, are consistent with the diagnosis of a Gartner cyst. No changes in the cyst have been found during follow-up to date.

### Case 4 – follow-up for 2 years

**Fig. 4:**
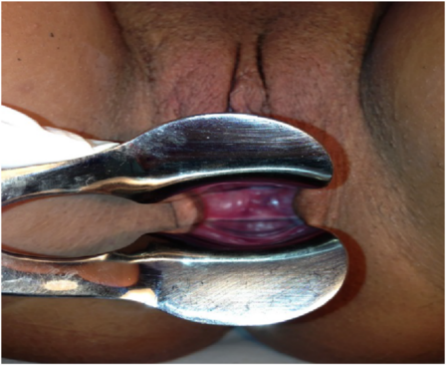
Cystic lesion of 2×2 cm in the right wall of the vagina

A 49-year-old woman of mixed ethnicity, para 2, was referred to our gynecological clinic because of a nontender mass inside her vagina. The mass had been evident for approximately 2 years, without any symptoms. She had a history of endometrial polyps and uterine fibroids. A pelvic examination revealed a 2 × 2 cm cystic lesion in the right wall of her vagina (Fig. 4). The mass was soft and could be compressed manually without difficulty. A pelvic ultrasound confirmed a cystic lesion in this region of her vagina. She was treated conservatively and had no interval cyst growth or change in cyst characteristics at follow-up.

## Discussion

This is one of the few studies of Gartner’s cysts with a series of consecutive cases followed over a long period of time who were exclusively subject to clinical observation. Most of the case series presented in the literature typically present patients who were treated surgically [8]. The follow-up of these patients ranged from 2 to 17 years. In these four cases the location of the cysts was the right wall of the vagina. Gartner’s cysts are typically small with an average diameter of 2 cm. However, these cysts can increase in size and be confused with other structures, such as cystocele and uterine prolapse [6]. In fact, in this series, the size ranged from 1.5 to 4.2 cm. In all cases, the diagnosis was made incidentally during routine pelvic examination and most lesions were asymptomatic. Only one of the four women had urethral stricture with a history of recurrent UTI, but this condition did not have any relation with the presence of the cyst (Case 3). Transvaginal ultrasound was the test of choice for diagnostic confirmation (Fig. 3b). The investigation of the characteristics of these lesions can be made with urinary tract imaging tests, such as ultrasound and MRI. Intravenous pyelography and computed tomography are additional examinations that may be requested [1]. Treatment depends on the symptoms and desire of the patient. In the cases presented in this study, the women were asymptomatic and chose to be observed clinically. In this situation, surgery is not typically performed because this type of surgery can be complex and is not recommended unless the patient has severe symptoms [9]. When a patient is symptomatic, the initial procedure can involve cyst drainage, injection, or aspiration, and intra-cystic tetracycline [10]. In large and symptomatic or recurrent cysts, excision or marsupialization is indicated [4]. Cyst marsupialization is a simple minimally invasive procedure, which creates minimal surgical scarring and results in the pathological diagnosis of a Gartner duct cyst. Long-term follow-up after such a procedure has proven its efficacy with no demonstrated side effects or recurrence [11]. The management strategies for multiloculated recurrences include periodic surveillance, sclerotherapy, and marsupialization [12]. In older patients, a mass wall biopsy is recommended to exclude neoplasia; however, malignant transformation of Gartner’s cysts is exceedingly rare [13].

## Conclusions

Despite the small number of cases of Gartner’s cysts, this study shows that conservative treatment can be a safe option for asymptomatic patients. However, further studies with a larger number of women in older age groups are needed for a better definition of conduct for these patients.
